# Reduction of amylose–amylopectin ratio in low-protein diets: impacts on growth performance and intestinal health in weaned pigs

**DOI:** 10.1093/jas/skae370

**Published:** 2024-12-09

**Authors:** Federico Correa, Diana Luise, Sara Virdis, Clara Negrini, Barbara Polimeni, Roxana Elena Amarie, Andrea Serra, Giacomo Biagi, Paolo Trevisi

**Affiliations:** Department of Agro-Food Sciences and Technologies, University of Bologna, Bologna, Italy; Department of Agro-Food Sciences and Technologies, University of Bologna, Bologna, Italy; Department of Agro-Food Sciences and Technologies, University of Bologna, Bologna, Italy; Department of Agro-Food Sciences and Technologies, University of Bologna, Bologna, Italy; Department of Agro-Food Sciences and Technologies, University of Bologna, Bologna, Italy; Department of Agricuture, Food and Environment, University of Pisa, Pisa, Italy; Department of Agricuture, Food and Environment, University of Pisa, Pisa, Italy; Department of Veterinary Science, University of Bologna, Ozzano dell’Emilia, Italy; Department of Agro-Food Sciences and Technologies, University of Bologna, Bologna, Italy

**Keywords:** nitrogen excretion, feed-grade amino acids, gut microbiota, waxy corn, postweaning diarrhea

## Abstract

Improving the synchrony between amino acids (**AAs**) and glucose appearance in the blood can support the growth performance of weaned pigs fed a low crude protein (**CP**) diet. This can be achieved using a diet with a low amylose-to-amylopectin ratio (**AM/AP**). The aim of this experiment was to evaluate whether reducing the AM/AP by using a corn variety characterized by a high amylopectin content, in the weaning diet can sustain growth performance and improve the intestinal health of pigs fed a low-CP diet. At weaning (25 ± 2 d), 90 pigs were assigned to 3 treatment groups: 1) control group (**CTR**), fed a standard diet with a medium-high CP content and high AM/AP (days 0 to 13: 18.0% CP, 0.13 AM/AP; days 14 to 27: 16.6% CP, 0.30 AM/AP; days 28 to 49: 16.7% CP, 0.15 AM/AP); 2) a group fed a low-CP diet with a high AM/AP (**LP**) (days 0 to 13: 16.0% CP, 0.17 AM/AP; days 13 to 27: 14.7% CP, 0.17 AM/AP; days 28 to 49: 14.5% CP, 0.25 AM/AP); 3) a group fed a low CP and a low AM/AP diet (**LPLA**) (days 0 to 13: 16.0% CP, 0.09 AM/AP; days 14 to 27: 14.7% CP, 0.05 AM/AP; days 28 to 49: 14.5% CP, 0.09 AM/AP). Pigs were weighted weakly until day 49. Fecal samples were collected on days 10 and 42 (12 samples/group/timepoint) for ammonia and calprotectin content and microbiota profile characterization. Until day 28, body weight (**BW**) of pigs from CTR was not different from pigs of the LPLA group, whereas it was higher from pigs of the LP group (*P* < 0.05). Thereafter, CTR group had greater BW compared with LP and LPLA groups for all the other timepoints considered (*P *< 0.05). From days 0 to 7 LPLA group had a lower incidence of diarrhea than the LP group (*P *= 0.04). On day 10, LPLA group had a greater alpha diversity (Shannon and InvSimpson indices), than the CTR (*P* = 0.03) and LP (*P* = 0.04) groups. On day 42, LPLA group had significantly greater InvSimpson diversity than LP group (*P *= 0.028). On day 10, LP group was characterized by greater abundance of *Lactobacillus* (LDA score = 5.15, *P *= 0.02), *Clostridium*-*sensu*-*stricto*-1 (LDA score = 4.90, *P *= 0.02) and Oscillospiraceae NK4A214-group (LDA score = 4.87, *P *= 0.004), whereas LPLA group was characterized by greater abundance of *Prevotella* (LDA score = 5.04, *P *= 0.003) and *Agathobacter* (LDA score = 4.77, *P *= 0.05). In conclusion, while reducing CP levels may negatively impact growth performance, when combined with higher amylopectin levels, it can reduce the incidence of diarrhea and increase fecal microbial diversity.

## Teaser

Results of the experiment demonstrated that a low-protein diet combined with a reduction in the amylose-to-amylopectin ratio supported pigs growth in the first 2 wk after weaning, decreased diarrhea, and increased their gut bacterial diversity.

## Introduction

In recent years, the topic of reducing crude protein (**CP**) in the diets of postweaning pigs has garnered significant interest. Lower CP diets offer notable advantages, such as reducing nitrogen excretion, which has a positive impact on the environmental footprint of livestock (Liu et al., 2017; Pomar et al., 2021), and reducing the risk of gut disorders (Yu et al., 2019; Limbach et al., 2021; Luise et al., 2021).

Over the past decades, the availability of feed-grade amino acids (**AAs**) has enabled more precise diet formulation, allowing for a reduction in dietary CP without compromising animal health and performance (Wang et al., 2018). However, reducing CP beyond a certain threshold, even when AAs requirements are met with feed-grade AAs, appears to negatively impact growth performance (Lynegaard et al., 2021). Nonetheless, replacing complex proteins with crystalline AAs raises concerns about absorption kinetics (Eugenio et al., 2022). Indeed, the AAs from complex protein sources are released more slowly than crystalline AAs, which are rapidly absorbed from the intestine to the bloodstream (Eugenio et al., 2022). While the concentration of free AAs in the blood stimulates protein synthesis, this process can be hindered if the AAs peak does not coincide with adequate glucose levels and a subsequent insulin spike, leading to AAs catabolism via hepatic deamination (Eugenio et al., 2023). Pigs typically derive glucose from starch digestion, but because starch digestion is slower compared with crystalline AAs absorption, this asynchrony can reduce the efficiency of AAs utilization, particularly in low-CP diets (Eugenio et al., 2023). To address this issue, optimizing the synchrony between glucose and AAs peaks in the bloodstream using fast-digestible starch may improve AAs utilization and sustain growth performance (Liu and Selle, 2017).

Starches rich in amylopectin (**AP**) increase blood glucose levels more rapidly than those based on amylose (**AM**) in growing pigs (Zhou et al., 2022). Amylopectin, being a branched polysaccharide, is rapidly degraded by α-amylase in the small intestine (Zhou et al., 2022). Consequently, raw materials high in AP content can be used to decrease the AM/AP ratio an thus to increase the blood glucose uptake (Martens et al., 2018). The use of cassava as an AP source in low-CP diets increased intestinal glucose uptake and gut hormone secretion, improving growth performance and nitrogen efficiency in growing pigs (Zhou et al., 2021). Moreover, Zhou et al. (2021) reported that combining corn starch and waxy corn starch (with a lower AM/AP ratio) enhanced whole-body protein deposition, growth performance, and reduced urinary nitrogen excretion in low-CP diets. Beyond growth performance, reducing dietary CP while increasing AP can influence the gut microbiota ecosystem, impacting various aspects of gut health (Ren et al., 2021). Indeed, lowering the CP level can reduce the amount of undigested protein reaching the hindgut, potentially decreasing the production of toxic compounds that adversely affect gut health (Luise et al., 2021). Conversely, higher AP content can alter digestive kinetics, reducing substrate availability for gut microbiota due to its higher enzymatic breakdown, which reduces fermentation compared with AM-rich starch, thus reducing short-chain fatty acid (**SCFA**) concentrations in the gut (Yang et al., 2021), crucial for gut epithelial cell energy. Therefore, we hypothesized that formulating a low-CP diet and improving the synchrony between blood AAs and glucose release can enhance protein efficiency without affecting the gut health of postweaning pigs.

This study aims to assess whether reducing the AM/AP ratio in a low-CP diet can maintain the growth performance and intestinal health of weaned pigs. The results were compared with a diet with conventional CP levels and AM/AP ratios, which served as a benchmark.

## Material and Methods

The animals enrolled in the present study were raised under the indication of the Dir. 120/2008 EC. The study was reviewed and approved by Ethical Committee of the University of Bologna (Protocol ID: 4416. Prot. n. 0007994 24/10/2022).

### Animals and experimental design

At weaning (25 ± 2 d old), 90 pigs were transferred from a commercial farm to the facility of the University of Bologna. Upon arrival, pigs were weighed and divided into 3 treatments group balanced for their body weight (**BW**) and litter of origin. Each group consisted of 10 experimental units (pens) of 3 pigs per pen.

The pens had mesh flooring and were equipped with enrichment materials, including a chain and a natural cotton rope. Pigs had unrestricted access to feed and water throughout the experimental period, with feed provided ad libitum. Room temperature was kept controlled from 30 °C at the start of the experiment to 25 °C at the end of the experiment, with a 1 °C decrease every 3 d. Each group was assigned to one of 3 experimental diets: 1) control group (**CTR**), fed a standard diet with a medium-high CP content and high AM/AP (days 0 to 13: 18.0% CP, 0.13 AM/AP; days 14 to 27: 16.6% CP, 0.30 AM/AP; days 28 to 49: 16.7% CP, 0.15 AM/AP); 2) a group fed a low-CP diet with high AM/AP (**LP**) (days 0 to 13: 16.0% CP, 0.17 AM/AP; days 14 to 27: 14.7% CP, 0.17 AM/AP; days 28 to 49: 14.5% CP, 0.25 AM/AP); 3) a group fed a low protein and low AM/AP (**LPLA**) (days 0 to 13: 16.0% CP, 0.09 AM/AP; days 14 to 27: 14.7% CP, 0.05 AM/AP; days 28 to 49: 14.5% CP, 0.09 AM/AP). Waxy corn, a corn cultivar, approved for animal feed, was used as the source of AP, with a starch that is more than 99% represented by AP. The composition of the diets, their chemical composition, and the amounts and ratio of AM to AP are reported in Table 1. After production, the AAs profile was analyzed in all diets (Table S1) and any AAs deficiency compared with the calculated AAs composition was corrected in the entire feed batch through the addition of crystalline AAs.

**Table 1.** Composition of the experimental diets

Item	Phase 1: days 0 to 13			Phase 2: days 14 to 27			Phase 3: days 28 to 49		
	CTR	LP	LPLA	CTR	LP	LPLA	CTR	LP	LPLA
Ingredients									
Bakery meal, %	18	18	18	17.4	20.4	19.8	—	—	—
Whey powder, %	10.8	9.4	9.4	5.6	5.6	5.6	—	—	—
Waxy corn, %	—	—	15	—	—	15	—	—	20
Barley, %	10	10	10	10	10	10	15	15	15
Wheat, %	10	10	10	8	10	10	10	10	10
Corn flaked, %	7	7	—	7	7	—	—	—	—
Wheat bran, %	6.4	6.1	6.1	3	6.9	6.5	—	—	—
Barley flaked, %	5	5	5	5	5	5	—	—	—
Wheat middlings, %	5	5	5	5	7	7	12.5	13.9	13.9
Soy protein concentrate^1^, %	3.9	—	—	7.7	—	—	5	—	—
Corn, %	3.2	8	—	13	7	—	36.9	41.2	21.2
Sunflower meal, %	3	3	3	3	3	3	—	—	—
Egg, Whole, Spray Dried, %	3	3	3	—	—	—	—	—	—
Premix 1^2^, %	2	2	2	2	2	2	1.9	1.9	1.9
Premix 2^3^, %	0.3	0.3	0.3	0.2	0.2	0.2	0.2	0.2	0.2
Premix minerals and vitamins^4^, %	0.5	0.5	0.5	0.5	0.5	0.5	0.4	0.4	0.4
Wheat gluten, %	2	1	1	1	1	1	—	—	—
Lysine premix^5^, %	2.3	3	3	2.4	3.4	3.4	2.5	3.4	3.4
Methionine premix^6^, %	1	2.1	2.2	0.1	0.3	0.2	0	0.2	0.2
Vegetable oil, %	1.8	1.5	1.5	1.4	1.8	1.8	0.6	—	—
Lignocellulose, %	1.5	1.5	1.5	1.5	1.5	1.5	—	—	—
Soybean meal, %	1	1	1	2	2	2	10.8	8.4	8.4
Dicalcium phosphate, %	0.7	0.8	0.8	0.8	0.8	0.8	0.6	0.7	0.7
Formic acid, %	0.7	0.7	0.7	0.7	0.7	0.7	—	—	—
SCFA/MCFA mixture^7^, %	0.4	0.4	0.4	0.3	0.3	0.3	0.2	0.2	0.2
Sodium chloride, %	0.2	0.2	0.2	0.3	0.3	0.3	0.7	0.7	0.7
Choline, %	0.2	0.2	0.2	0.2	0.2	0.2	0.1	0.1	0.1
Mycotoxin binder^8^, g/kg	0.1	0.1	0.1	0.1	0.1	0.1	0.1	0.1	0.1
Phytase, 5,000 FTU/g, %	0.1	0.1	0.1	0.1	0.1	0.1	0.1	0.1	0.1
Calcium carbonate, %	—	—	—	—	—	—	0.3	0.3	0.3
L-Tryptophan^8^, g/kg	0.13	0.45	0.43	0.01	0.01	0.04	0.01	0.04	0.05
L- Valine^8^, g/kg	0.57	1.26	1.36	0.13	0.2	0.23	0.02	0.14	0.13
L-Threonine^8^, g/kg	0.55	1.15	1.31	0.03	0.1	0.15	0.02	0.1	0.11
Histidine HCL^8^, g/kg	0.27	0.52	0.52	0.02	0.07	0.06	0.01	0.07	0.07
Isoleucine^8^, g/kg	0.61	1.3	1.34	0.03	0.15	0.14	0.02	0.14	0.14
L-Leucine^8^, g/kg	0.96	2.07	2.17	0.07	0.27	0.23	0.04	0.21	0.19
L-Lysine^8^, g/kg	0.52	0.22	0.92	0.03	0.05	0.01	0.04	0.05	0.04
L-Methionine^8^, g/kg	0.12	0.3	0.34	0.08	0.04	0.04	0.03	0.05	0.02
Nutrients composition, %									
Starch^9^	31.4	36.6	34.2	34.7	38.7	38.2	40.5	41.8	42.4
Amylose^9^	3.5	5.2	2.8	8.1	5.5	1.7	5.3	5.3	3.5
Amylopectin^9^	27.9	31.4	31.4	26.6	33.2	36.5	35.2	36.5	38.9
Amylose/amylopectin ratio^9^	0.1	0.2	0.1	0.3	0.2	0.1	0.2	0.2	0.1
Crude protein	18	16	16	16.6	14.7	14.7	16.7	14.5	14.5
Crude lipids	6.4	6.1	6.1	5.2	5.9	5.8	3.1	2.7	2.7
Crude fiber	4.4	4.4	4.4	4.4	4.6	4.6	3.2	3.2	3.2
Ash	4.7	4.5	4.5	4.7	4.6	4.6	5	4.7	4.7
SID Lysine	1.14	1.14	1.14	1.08	1.08	1.08	1.13	1.13	1.13
SID Methionine	0.38	0.41	0.41	0.39	0.41	0.41	0.42	0.45	0.45
SID Tryptophan	0.27	0.28	0.28	0.25	0.25	0.25	0.25	0.25	0.25
SID Valine	1.14	1.14	1.14	1.08	1.08	1.08	1.13	1.13	1.13
SID Isoleucine	0.63	0.63	0.63	0.56	0.56	0.56	0.56	0.56	0.56
SID Leucine	1.16	1.16	1.16	1.08	1.08	1.08	1.13	1.13	1.13
SID Histidine	0.34	0.34	0.34	0.34	0.34	0.34	0.37	0.37	0.37
Net energy, Mcal/kg	2.40	2.40	2.40	2.40	2.30	2.40	2.30	2.30	2.30

Abbreviations: CTR = control group fed a standard diet with a medium-high CP content and high AM/AP (d 0 to 13: 18.0% CP, 0.13 AM/AP; days 14 to 28: 16.6% CP, 0.30 AM/AP; days 28 to 49: 16.7% CP, 0.15 AM/AP), LP = group fed a low-CP diet with high AM/AP (days 0 to 14: 16.0% CP, 0.17 AM/AP; days 14 to 28: 14.7% CP, 0.17 AM/AP; days 28 to 49: 14.5% CP, 0.25 AM/AP), LPLA = group fed a low protein and low AM/AP (days 0 to 14: 16.0% CP, 0.09 AM/AP; days 14 to 28: 14.7% CP, 0.05 AM/AP; days 28 to 49: 14.5% CP, 0.09 AM/AP).

^1^2,82% SID Lysine.

^2^Premix provided the following per kilogram of diet: L-Tryptophane 42,500 mg, L-Valine 66,000 mg, L-Threonine 130,000 mg, endo-1,4-beta-glucanase 4,000 U, endo-1,3(4)- beta-glucanase 3,500 U, endo-1,4-beta-xylanase 13,500 U, *S. cerevisiae* 10^9^ CFU.

^3^Premix provided the following per kilogram of premix: citric acid 150 g, sorbic acid 167 g, thymol 17 g, vanillin 10 g.

^4^Premix provided the following per kilogram of premix: Fe 30,000 mg, Mn 16,000 mg, Zn 8,000 mg, Cu 20,000, I 360 mg, Se 60 mg, Vit. A 3,200,000 UI, Vit. D3 400,000 UI, Vit. E (all-rac) 30,000 UI, Vit. K3 612 mg, Vit B1 1,000 mg, Vit. B2 2,000 mg, Vit. B6 1,400 mg, Vit B12 20 mg, Biotine 20 mg, Nicinamide 12,000 mg, Folic Acid 300 mg, Calcium D-pantothenate 4,000 mg, vit. C 28,000 mg.

^5^Premix composition: 25 % of Lysine HCL (78%) and 75% of wheat middling.

^6^Premix composition: 10% of DL-Methionine (99%) and 90% of wheat middling.

^7^Selection of α-monoglycerides of short- and medium-chain fatty acids (C1, C3, C4, C6, C8, C10).

^8^This ingredients were added on top of the final diet.

^9^Amylose and Amylopectin are analyzed values: others were calculated.

### Sampling

Pigs were individually weighed on day 0 (day of weaning) and then weekly until day 49. Feed consumption and residues were measured weekly in order to calculate the gain-to-feed ratio (**G:F**). Health status was monitored daily and fecal consistency was assessed by applying a 5-point scale (1: hard feces to 5: watery feces), as described by Correa et al. (2022), diarrhea was declared when fecal score was higher than 3.5. On days 10 and 42, from a subgroup of 12 pigs per group (72 samples in total), a rectal swab was taken and snap frozen in liquid nitrogen. These samples were used for gut microbiota profile, SCFAs, and ammonia concentration.

### Amylose and amylopectin quantification

An adequately homogenized feed sample aliquot was carefully weighed and subjected to the extraction process. The starch within the samples was dispersed by heating in dimethyl sulfoxide. Lipids were eliminated by precipitating the starch in ethanol and subsequently recovering the precipitated starch. Following the dissolution of the precipitated sample in an acetate/salt solution, AP was selectively precipitated with the addition of concanavalin A and subsequently separated via centrifugation. Amylose, present in an aliquot of the supernatant, was enzymatically hydrolyzed to D-glucose, which was later analyzed using the glucose oxidase/peroxidase reagent. Total starch in a separate aliquot of the acetate/salt solution was similarly hydrolyzed to D-glucose and quantified through colorimetric measurements using glucose oxidase/peroxidase. The concentration of AM in the starch sample was estimated by calculating the ratio between the absorbance of glucose oxidase/peroxidase at 510 nm in the supernatant of the Concanavalin A-precipitated sample and that of the total starch sample.

### SCFAs and ammonia in feces

The quantification of SCFAs such as acetate, propionate, isobutyrate, butyrate, valerate, and isovalerate, along with lactic acid, in fecal samples was conducted using High-Performance Liquid Chromatography. The process involved diluting 5 g of feces in 25 mL of 0.1 N H_2_SO_4_ aqueous solution, followed by a 2-min homogenization using an UltraTurrax (IKA-Werke GmbH & Co. KG, Staufen, Germany). Post homogenization, the mixture underwent centrifugation at 5,000 × g for 15 min at 4 °C to segregate the liquid phase from the solid residue. This was then microfiltered using a 0.45-μm Millex-HV filter (Merck-Millipore, Billerica, MA). The resultant sample was directly injected into the chromatograph, equipped with an Aminex 85 HPX-87 H ion exclusion column (300 mm × 7.8 mm; 9 μm particle size; Bio-Rad, Milan, Italy), maintained at 40 °C. Detection occurred at a wavelength of 220 nm. The analysis involved isocratic elution at a flow rate of 0.6 mL/min using a 0.008 N H_2_SO_4_ solution as the mobile phase and a 20 μL injection loop. The SCFAs and lactic acid were identified using a standard solution comprising various concentrations of acids in 0.1 N H_2_SO_4_ (Sigma-Aldrich, Milan, Italy). Quantification was based on an external calibration curve created from external standards as described by Sandri et al. (2017).

In the determination of ammonia in fecal samples, the samples were first thawed. Then, 1 g of feces was diluted in a 1:10 weight/volume ratio with deionized water. After vortexing, the samples were centrifuged for 10 min at 7,000 rpm and 4 °C. The ammonia content in the fecal, supernatant was assessed using an enzymatic colorimetric assay, adhering to the manufacturer’s protocol (Urea/BUN-Color; BioSystems S.A., Barcelona, Spain), and the results were reported in μmol/g of feces.

### Fecal calprotectin

Fecal calprotectin concentration (ng/mL) was analyzed using the Enzyme-Linked Immunosorbent Assay kit (MBS033848, Mybiosource, San Diego, CA, USA) following the supplier’s instructions. Before analysis, fecal, samples were diluted 1:70 in a phosphate buffered saline solution. The samples were analyzed in duplicate. The absorbance of the samples was read at 450 nm using Multiskan FC Microplate Photometer Multiplate Reader (Thermo Fisher Scientific, USA). Calprotectin concentration was calculated using a 4-point parametric curve.

### Microbial profile

Bacterial DNA extraction in 72 fecal samples was carried out using the FastDNA SPIN Kit for Soil (MP Biomedicals, Santa Ana, Ca, USA), strictly adhering to the guidelines provided by the manufacturer. The concentration and purity of the extracted DNA were evaluated using NanoDrop spectrophotometry (Fisher Scientific, 13 Schwerte, Germany), focusing on absorbance ratios at 260/280 and 260/230 to assess purity. For the amplification of the V3-V4 region of the 16S rRNA gene, approximately 460 base pairs in length, universal primers Pro341F (5ʹ-TCGTCGGCAGCGTCAGATGTGTATAAGACCCTACGGGNBGCASCAG-3ʹ) and Pro805R (5ʹGTCTCGTGGCTCGGAGATGTGTATAAGACAGGACTACNVGGTATCTAATCC-3ʹ) as reported by Takahashi et al. (2014) were employed. This amplification was performed using Platinum™ Taq DNA Polymerase High Fidelity (Thermo Fisher Scientific, Italy). Sequencing of the resulting amplicons was conducted on the Illumina MiSeq 300 × 2 bp platform. The creation of the library and the sequencing of the 16S rRNA gene were executed using the MiSeq Reagent Kit V3-V4 on the MiSeq-Illumina platform. For the analysis of the microbiota, the DADA2 pipeline (Callahan et al., 2016) was utilized, and for taxonomic assignments, the Silva Database (release 138.1) was used as a reference, as described by Quast et al. (2013).

### Statistical analysis

A linear mixed model that considered diet as a fixed factor and litter of origin as a random factor was used for statistical analysis of BW, average daily gain (**ADG**), SCFAs, NH_3_, and calprotectin data. For feed intake and G:F, the pen was used as the experimental unit and the data were fitted with a linear model that included diet as a fixed factor. An ANOVA test was used in conjunction with these models to test for statistically significant differences among the dietary treatments. Comparisons among diets were tested with a post hoc test (Tukey test). Diagnostic plots, such as normal Q-Q plots, were utilized to visually assess the distribution of the model residuals and ensure adherence to the normality assumption. Additionally, formal statistical tests, such as the Shapiro–Wilk test, were employed to quantitatively evaluate the normality of the data. Statistical analyses were performed using the functions in the “car”,”lsmeans”, and “lme4” packages within the R v4.1.1 software.

For the microbiota data, the statistical analysis on alpha and beta diversity and taxonomic differences was performed with R v4.1.1, using “phyloseq” v1.38 (McMurdie and Holmes, 2013), “vegan” v2.6 (Dixon, 2003) and “microbiomeutilities” v1.0. Sample abundances were normalized by rarefaction with reference to the sample with the lowest number of sequences, to avoid bias related to the different number of sequences produced per sample. Between-group differences in alpha diversity indices (Chao1, Shannon, and Simpson diversity) were tested using the Wilcoxon test.

For beta diversity, a dissimilarity matrix was constructed using as a metric the Euclidean distance between the abundances of the transformed samples using the “clr” transformation; the results were plotted using a nonmetric multidimensional scaling plot. Differences were tested using a PERMANOVA (Adonis) model with 9,999 permutations, including diet as a factor.

For differential analysis of taxa, the LEfSe algorithm (Segata et al., 2011) was used at the genus level (LDA score > 3 and *P* < 0.05) among experimental diets. R scripts used for the statistical and bioinformatic analysis are reported in the Supplementary Material.

Raw sequence data are freely available at NCBI Sequence Read Archive under the accession number PRJNA1055475.

## Results

### Growth performance

The results of individual growth performance are shown in Table 2. Diet had a significant effect on BW on days 21 (*P *= 0.01), 28, 35, 42, and 49 (*P *< 0.05). On days 21 and 28, the CTR group had a greater BW than LP group (*P *= 0.01 and *P *< 0.05, respectively), whereas CTR and LPLA groups were not different. On days 35, 42, and 49, the CTR group had a greater BW than the LP group (*P *< 0.05) and LPLA group (*P *< 0.05), and there were no differences between the LP and LPLA groups. Diet significantly influenced ADG considering the periods: days 14 to 28 (*P *< 0.05), days 28 to 42 (*P *< 0.05), and days 0 to 49 (*P *< 0.05) whereas a trend was observed between days 0 and 14 *(P *= 0.06). Pairwise contrast evidenced the CTR group had greater ADG than the LP group for the periods: days 14 to 28 (*P *< 0.05), days 28 to 35 (*P *< 0.05), days 35 to 42 (*P *< 0.05), days 28 to 49 (*P *< 0.05), and days 0 to 49 (*P *< 0.05), whereas a trend was observed for days 0 to 14 (*P *= 0.06). In addition, the CTR group had greater ADG than the LPLA group in the periods: days 14 to 28 (*P *< 0.05), days 28 to 49 (*P *< 0.05), and days 0 to 49 (*P *< 0.05). No differences were observed between LP and LPLA groups in all periods considered.

**Table 2.** Effect of CP level and different amylose/amylopectin ratio in piglets’ diets on BW, daily weight gain, feed intake and gain-to-feed ratio in the weaning phase

Item	Diet			SEM	*P*-value
	CTR	LP	LPLA		
BW (kg)					
Day 0	6.9	6.9	6.9	0.3	0.95
Day 7	7.7	7.6	7.6	0.3	0.98
Day 14	9.7	9.4	9.6	0.3	0.48
Day 21	12.8^a^	11.6^b^	12.1^ab^	0.4	<0.05
Day 28	16.0^a^	14.5^b^	15.1^ab^	0.4	<0.05
Day 35	20.0^a^	17.8^b^	18.1^b^	0.5	<0.05
Day 42	24.5^a^	21.6^b^	22.3^b^	0.5	<0.05
Day 49	31.2^a^	27.4^b^	28.1^b^	0.6	<0.05
Average daily gain (g/d)					
Days 0 to 14	200	175	192	10.3	0.03
Days 14 to 28	466^a^	366^b^	392^b^	16.3	<0.05
Days 28 to 49	725^a^	615^b^	620^b^	16.6	<0.05
Days 0 to 49	495^a^	419^b^	433^b^	10.7	<0.05
Feed intake (g/d)					
Days 0 to 14	269.3	276.5	299.7	11.4	0.16
Days 14 to 28	682.7	659.8	691.5	15.6	0.35
Days 28 to 49	1,097.2	1,049.3	1,041.1	31.5	0.18
Days 0 to 49	733.1	750.2	706.4	13.2	0.07
Gain:feed					
Days 0 to 14	0.74	0.63	0.64	0.08	0.5
Days 14 to 28	0.68^a^	0.55^b^	0.57^b^	0.06	<0.05
Days 28 to 49	0.66^a^	0.59^b^	0.60^b^	0.07	<0.05
Days 0 to 49	0.68	0.64	0.64	0.05	0.8

Abbreviations: CTR = control group fed a standard diet with a medium-high CP content and high AM/AP (days 0 to 13: 18.0% CP, 0.13 AM/AP; days 14 to 28: 16.6% CP, 0.30 AM/AP; days 28 to 49: 16.7% CP, 0.15 AM/AP), LP = group fed a low-CP diet with high AM/AP (days 0 to 14: 16.0% CP, 0.17 AM/AP; days 14 to 28: 14.7% CP, 0.17 AM/AP; days 28 to 49: 14.5% CP, 0.25 AM/AP), LPLA = group fed a low protein and low AM/AP (days 0 to 14: 16.0% CP, 0.09 AM/AP; days 14 to 28: 14.7% CP, 0.05 AM/AP; days 28 to 49: 14.5% CP, 0.09 AM/AP).

^a,b^Values within a row with different superscripts differ significantly at *P *< 0.05.

The CTR group had a lower G:F than LP and LPLA group considering the periods days 14 to 28 (*P *< 0.05 and *P *< 0.05, respectively), and days 28 to 49 (*P *< 0.05 and *P *< 0.05, respectively).

Results on the fecal, index are shown in Table 3. LPLA group had a lower fecal, index than the LP group (*P *< 0.05). No significant differences were observed for the period days 7 to 14. No diarrhea events were registered after day 14.

**Table 3.** Effect of CP level and different amylose/amylopectin ratio in piglets’ diets on diarrhea index in the weaning phase

	Diet				
Item	CTR	LP	LPLA	SEM	*P*-value
Fecal index (number of days with diarrhea > 3, %)^1^					
Days 0 to 7	0.30^ab^	0.53^a^	0.13^b^	0.3	<0.05
Days 7 to 14	0.03	0.23	0.17	0.06	0.07

Abbreviations: CTR = control group fed a standard diet with a medium-high CP content and high AM/AP (days 0 to 13: 18.0% CP, 0.13 AM/AP; days 14 to 28: 16.6% CP, 0.30 AM/AP; days 28 to 49: 16.7% CP, 0.15 AM/AP), LP = group fed a low-CP diet with high AM/AP (days 0 to 14: 16.0% CP, 0.17 AM/AP; days 14 to 28: 14.7% CP, 0.17 AM/AP; days 28 to 49: 14.5% CP, 0.25 AM/AP), LPLA = group fed a low protein and low AM/AP (days 0 to 14: 16.0% CP, 0.09 AM/AP; days 14 to 28: 14.7% CP, 0.05 AM/AP; days 28 to 49: 14.5% CP, 0.09 AM/AP).

^1^No diarrhea registered after day 14.

^a,b^Values within a row with different superscripts differ significantly at *P* < 0.05.

### Ammonia, calprotectin, and SCFAs

Results for ammonia, calprotectin and SCFAs concentrations in feces are shown in Table 4 and Table 5, respectively. No effect of the experimental diets on fecal, ammonia and calprotectin concentrations was observed on days 10 and 42.

**Table 4.** Effect of CP level and different amylose/amylopectin ration in piglets’ diets on fecal ammonia and calprotectin on days 10 and 42

Item	Diet				
	CTR	LP	LPLA	SEM	*P*-value
NH_3_, µmol/g					
Day 10	30.6	32.8	30.8	3.47	0.87
Day 42	36.4	27	35.8	3.85	0.12
Calprotectin, ng/mL					
Day 10	5,784	5,845	5,791	248	0.97
Day 42	5,007	5,139	5,387	297	0.62

Abbreviations: CTR = control group fed a standard diet with a medium-high CP content and high AM/AP (days 0 to 13: 18.0% CP, 0.13 AM/AP; days 14 to 28: 16.6% CP, 0.30 AM/AP; days 28 to 49: 16.7% CP, 0.15 AM/AP), LP = group fed a low-CP diet with high AM/AP (days 0 to 14: 16.0% CP, 0.17 AM/AP; days 14 to 28: 14.7% CP, 0.17 AM/AP; days 28 to 49: 14.5% CP, 0.25 AM/AP), LPLA = group fed a low protein and low AM/AP (days 0 to 14: 16.0% CP, 0.09 AM/AP; days 14 to 28: 14.7% CP, 0.05 AM/AP; days 28 to 49: 14.5% CP, 0.09 AM/AP).

^a,b^Values within a row with different superscripts differ significantly at *P* < 0.05.

**Table 5.** Effect of CP level and different amylose/amylopectin ratio in piglets’ diets on fecal concentration of lactic, acetic, propionic, isobutyric, butyric, valeric, and isovaleric acids on days 10 and 42

SCFAs (mmol/g)^1^	Diet				
	CTR	LP	LPLA	SEM	*P*-value
Day 10					
Lactic acid	0.016	0.013	0.016	0.003	0.69
Acetic acid	0.011	0.010	0.012	0.002	0.84
Propionic acid	0.011	0.007	0.012	0.003	0.43
Isobutyric acid	0.002	0.001	0.002	0.001	0.41
Butyric acid	0.005	0.003	0.004	0.001	0.43
Isovaleric acid	0.004	0.004	0.003	0.001	0.60
Valeric acid	0.001	0.003	0.001	0.001	0.10
Day 42					
Lactic acid	0.023	0.021	0.030	0.007	0.49
Acetic acid	0.023	0.026	0.021	0.005	0.72
Propionic acid	0.013	0.010	0.023	0.006	0.28
Isobutyric acid	0.001	<0.001	0.001	0.000	0.05
Butyric acid	0.008	0.020	0.013	0.006	0.14
Isovaleric acid	0.009	0.004	0.005	0.002	0.27
Valeric acid	0.002	0.002	0.002	0.000	0.86

Abbreviations: CTR = control group fed a standard diet with a medium-high CP content and high AM/AP (days 0 to 13: 18.0% CP, 0.13 AM/AP; days 14 to 28: 16.6% CP, 0.30 AM/AP; days 28 to 49: 16.7% CP, 0.15 AM/AP), LP = group fed a low-CP diet with high AM/AP (days 0 to 14: 16.0% CP, 0.17 AM/AP; days 14 to 28: 14.7% CP, 0.17 AM/AP; days 28 to 49: 14.5% CP, 0.25 AM/AP), LPLA = group fed a low protein and low AM/AP (days 0 to 14: 16.0% CP, 0.09 AM/AP; days 14 to 28: 14.7% CP, 0.05 AM/AP; days 28 to 49: 14.5% CP, 0.09 AM/AP).

^1^Data are expressed as mmol/g of fresh fecal, material.

^a,b^Values within a row with different superscripts differ significantly at *P *< 0.05.

For SCFAs concentration in feces, no effect of diet was detected on day 10, whereas a significant effect on isobutyric acid was detected on day 42 (*P *= 0.05). Pairwise contrast showed a tendency for greater isobutyric acid concentration in the feces of CTR pigs compared with LP pigs (*P *= 0.09).

### Fecal, microbial profile

Bacterial DNA from fecal, samples was successfully extracted and amplified from a total of 72 samples. In total, the sequencing procedure produced a total of 3,304,583 sequences; an average of 34,423 sequences per sample were retained after quality control. After bioinformatic analysis, a total of 4,679 Amplicon Sequence Variants (**ASVs**) were produced. The rarefaction curves in Figure S1 show the number of different species observed as a function of the number of sequences; the trend to a plateau indicates that the sequencing procedure was able to capture all the variability present in the samples.

Among the 4,679 ASVs recovered, 19 Phyla, 92 Families, and 293 Genera were identified. The most abundant phyla were Firmicutes 62.5 ± 8.4%, Bacteroidota,28.3 ± 7.0% and, Proteobacteria 1.6 ± 3.6%. The most abundant families were Prevotellaceae 19.5 ± 7.5%, Lachnospiraceae 14.5 ± 7.1%, Lactobacillaceae 9.5 ± 10.3% and Clostridiaceae 6.6 ± 6.7%. The most represented genera were *Prevotella* 11.2 ± 6.8%, *Lactobacillus* 9.45 ± 10.3%, *Clostridium-sensu-stricto-*1 6.39 ± 6.6% and Prevotellaceae-NK3B31-group 4.7 ± 4.4%.

**Figure 1.**
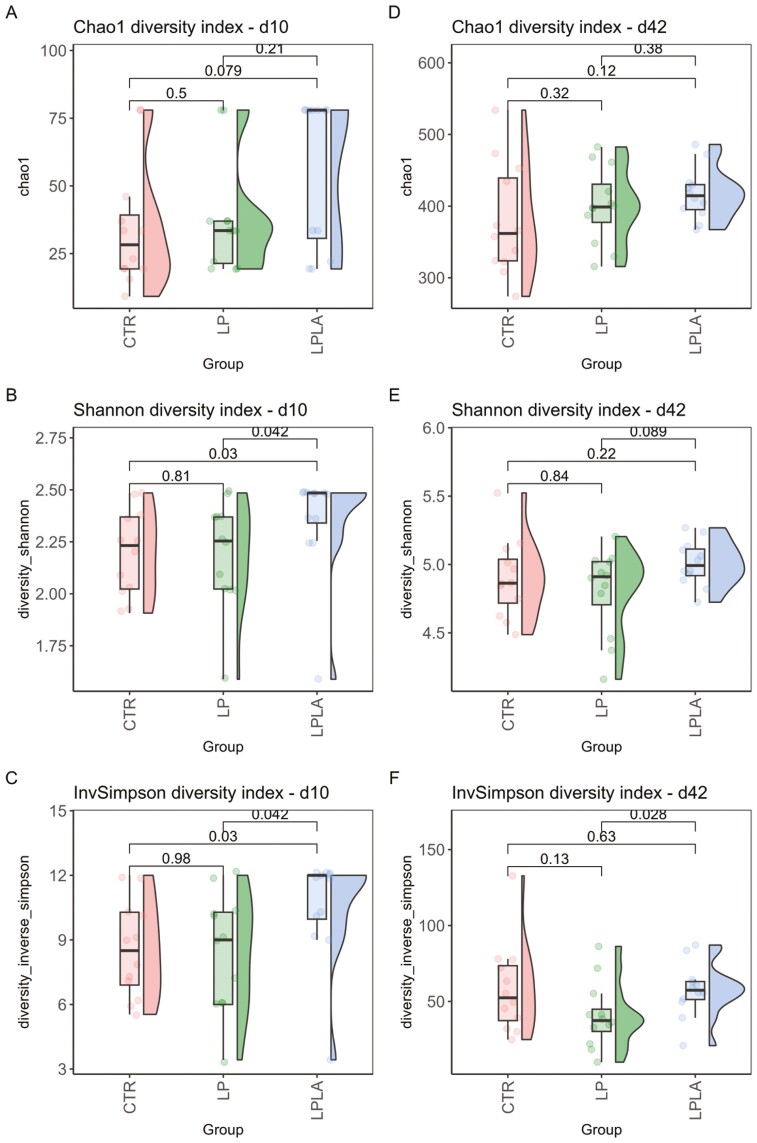
Effect of CP level and different amylose/amylopectin ratio in piglets diets on fecal alpha diversity on days 10 (A, B, and C) and 42 (D, E, and F) postweaning. ^1^CTR = control group fed a standard diet with a medium-high CP content and high AM/AP (days 0 to 13: 18.0% CP, 0.13 AM/AP; days 14 to 28: 16.6% CP, 0.30 AM/AP; days 28 to 49: 16.7% CP, 0.15 AM/AP), LP = group fed a low-CP diet with high AM/AP (days 0 to 14: 16.0% CP, 0.17 AM/AP; days 14 to 28: 14.7% CP, 0.17 AM/AP; days 28 to 49: 14.5% CP, 0.25 AM/AP), LPLA = group fed a low protein and low AM/AP (days 0 to 14: 16.0% CP, 0.09 AM/AP; days 14 to 28: 14.7% CP, 0.05 AM/AP; days 28 to 49: 14.5% CP, 0.09 AM/AP).

Figure 1 shows the values of Chao1, Shannon, and InvSimpson diversity indices for each group on days 10 and 42. Overall, on day 10, the LPLA group had a greater value of alpha diversity, as measured by Shannon and InvSimpson indices, than the CTR (*P <* 0.05) and LP (*P *< 0.05) groups. Whereas for the Chao1 index, the LPLA group tended to have a greater richness compared with the CTR group (*P *= 0.08). On day 42, the LPLA group tended to have a greater Shannon index than the LP group (*P *= 0.09) and significantly greater InvSimpson index than the LP group (*P *< 0.05). No significant differences were shown for Chao1 index and other comparisons on day 42.

**Figure 2.**
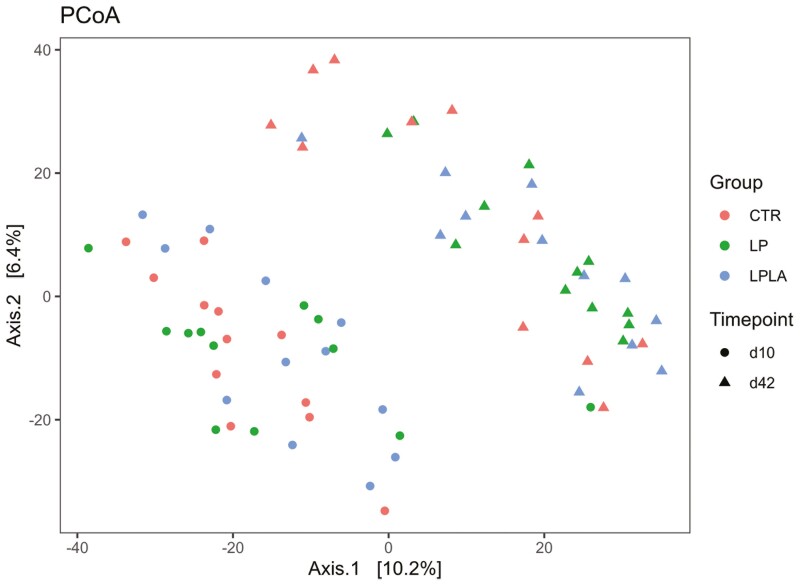
Effect of CP level and different amylose/amylopectin ratio in piglets’ diets on fecal beta diversity on days 10 and 42 postweaning. ^1^CTR = control group fed a standard diet with a medium-high CP content and high AM/AP (days 0 to 13: 18.0% CP, 0.13 AM/AP; days 14 to 28: 16.6% CP, 0.30 AM/AP; days 28 to 49: 16.7% CP, 0.15 AM/AP), LP = group fed a low-CP diet with high AM/AP (days 0 to 14: 16.0% CP, 0.17 AM/AP; days 14 to 28: 14.7% CP, 0.17 AM/AP; days 28 to 49: 14.5% CP, 0.25 AM/AP), LPLA = group fed a low protein and low AM/AP (days 0 to 14: 16.0% CP, 0.09 AM/AP; days 14 to 28: 14.7% CP, 0.05 AM/AP; days 28 to 49: 14.5% CP, 0.09 AM/AP).

For Beta diversity, 2 NMDS plots were generated, covering the two sampling point, using a Unifrac distance matrix (Figure 2). The plots showed a clear separation among the samples due to the timepoint (day 10 or day 42) whereas no clear separation among the samples of the different groups was observed, indicating that the overall microbial composition among the diets was not different. This is also evidenced by the Adonis test, which showed that the beta diversity was not affected by diet, both on day 10 (*P *= 0.72; R^2^ = 0.05,) and day 42 (*P *= 0.30; R^2^ = 0.06).

**Figure 3.**
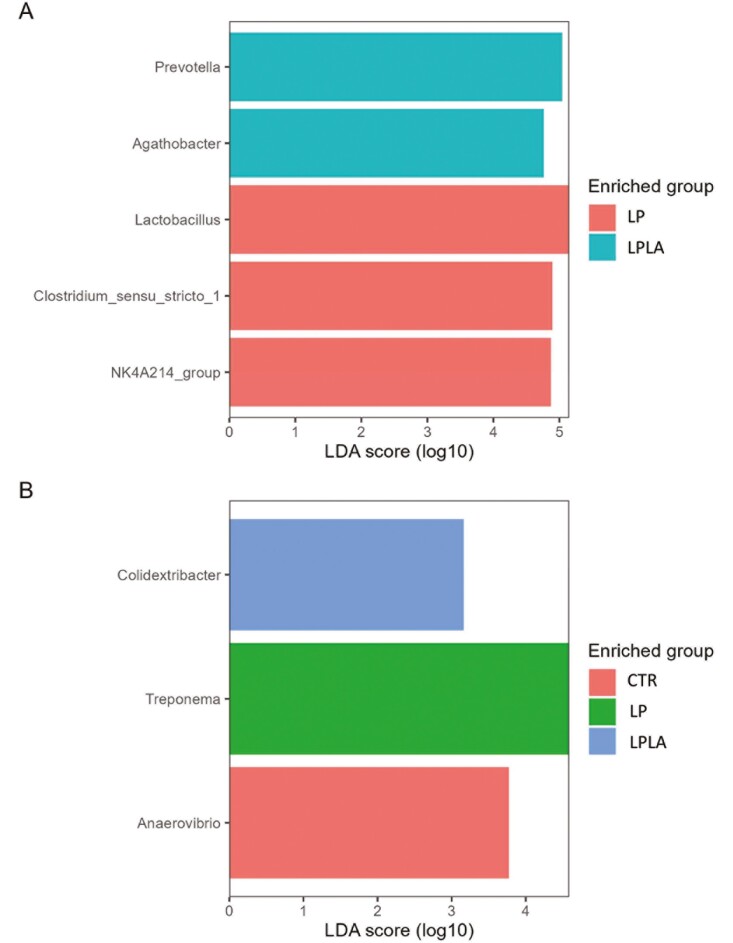
Effect of CP level and different amylose/amylopectin ratio in piglets’ diets on fecal microbial biomarkers at genus level on days 7 (A) 28 (B) postweaning. ^1^CTR = control group fed a standard diet with a medium-high CP content and high AM/AP (days 0 to 13: 18.0% CP, 0.13 AM/AP; days 14 to 28: 16.6% CP, 0.30 AM/AP; days 28 to 49: 16.7% CP, 0.15 AM/AP), LP = group fed a low-CP diet with high AM/AP (days 0 to 14: 16.0% CP, 0.17 AM/AP; days 14 to 28: 14.7% CP, 0.17 AM/AP; days 28 to 49: 14.5% CP, 0.25 AM/AP), LPLA = group fed a low protein and low AM/AP (days 0 to 14: 16.0% CP, 0.09 AM/AP; days 14 to 28: 14.7% CP, 0.05 AM/AP; days 28 to 49: 14.5% CP, 0.09 AM/AP).

To identify diet-specific bacterial markers, LEfSe analysis was carried out at both days 10 and 42 (Figure 3). On day 10, the LP group was characterized by a greater abundance of *Lactobacillus* (LDA score = 5.15, *P *< 0.05), *Clostridium-sensu-stricto-1* (LDA score = 4.90, *P *< 0.05) and Oscillospiraceae NK4A214-group (LDA score = 4. 87, *P *< 0.05) and the LPLA group was characterized by a greater abundance of *Prevotella* (LDA score = 5.04, *P *< 0.05) and *Agathobacter* (LDA score = 4.77, *P *< 0.05). On day 42, the CTR group was characterized by a greater abundance of *Anaerovibrio* (LDA score = 3.77, *P *< 0.05), the LP group by a greater abundance of *Treponema* (LDA score = 4.58, *P *< 0.05) and the LPLA group by a greater abundance of *Colidextribacter* (LDA score = 3.16, *P *< 0.05).

## Discussion

The use of a low-protein diet, combined with a reduced AM/AP ratio, did not negatively affect the animals’ weight up to the fourth week postweaning. However, beyond this period, the AM/AP ratio reduction was insufficient to maintain the weight of the animals.

To the authors’ knowledge, this is the first study reporting the results of low AM/AP ratio in low-CP diets in weaned pigs. A previous study by Zhou et al. (2022), in which different AM/AP ratios were tested in growing pigs fed a low-CP diet (~ 13%), demonstrated that an AM/AP ratio of ~0.2 provided the optimal dietary glucose release in vitro, increasing whole-body protein deposition and improving nitrogen efficiency and growth performance. However, growing pigs, are generally fed diets with lower dietary CP levels, and a lower amount of crystalline AAs is needed to balance the nutritional level of this diet compared with weaned pigs diet. Indeed, the amount of AAs in this study was ~ 2 g/100 g, which is particularly lower compared with the amount used in our study (~ 13 g/100g; in the first phase LPLA diet). This suggests that the AAs blood peak can be more pronounced in weaned pigs compared with growing pigs. Consequently, weaned pigs may benefit from a lower AM/AP, allowing for a faster appearance of glucose in the blood (Li et al., 2024), thereby optimizing protein synthesis. In this regard, data obtained in weaned pigs fed a diet with an AM/AP of ~0.03, compared with a diet with an AM/AP ratio of ~0.3 showed a positive effect on the ADG and G:F (Perez and Aumaitre, 1979). However, compared with the present study, these authors used a relatively high dietary CP level (20%), and no crystalline AAs were provided, which limits the comparability between the studies. Indeed, in our study, a strong restriction of the dietary CP (~ 14.6%) compensated by a high level of crystalline AAs was adopted. This solution is scarcely explored in weaned pigs, but it can contribute to explain the negative impact of the low-CP diets (both conventional and low AM/AP ratio diets) on the ADG and BW. This result agrees with other studies (Heo et al., 2008; Luo et al., 2015; Yu et al., 2019) in which the CP level was reduced to 15% or less. For instance, Yu et al. (2019) (20% vs 15%) and Luo et al. (2015) (20% vs 14%) observed a decrease in ADG in weaned pigs even though the diets were adjusted to meet the nutritional requirements with essential AAs. This suggests that non-essential AAs and/or other nutrients may have been deficient when CP was reduced below a certain threshold. In fact, it has been suggested that the combination of reducing the dietary CP and subsequently adjusting for the essential AAs profile may exacerbate the deficiency of non-essential AAs (Boisen et al., 2000), which may become limiting for certain biological functions, compromising protein synthesis. This is further supported by the observation that, according to NRC recommendations, the total nitrogen in the LP and LPHA diets, particularly in Phase 2 and Phase 3, was lower than the suggested SID total nitrogen values for pigs weighing 11 to 25 kg (2.35% vs. 2.56%). On the other hand, the lack of differences observed between the CTR and LPHA groups during the trial could be attributed to the fact that the CTR diet included a %7 of corn that was steam-flaked. This process leads to a gelatinizing of the starch granules and significantly modifies its structure. This alteration enhances digestibility, potentially providing a more readily available source of glucose (Xu et al., 2019), which may have helped pig of the CTR group to achieve a similar growth compared to the pigs of the LPHA group.

Apart from the effect on the growth performance, another effect of reducing dietary CP may be related to the modification of the substrates available for bacterial fermentation. In fact, it is plausible that reducing the amount of undigested protein or peptides that reach the large intestine may reduce the risk for toxic compound production like polyamines and phenolic compounds, thus preventing the increase in intestinal mucosa permeability that is associated with gut health disorders in weaned pigs (Gilbert et al., 2018). In this regard, the evaluation of well-targeted endpoints is of key importance for the evaluation of the dietary strategies in animal models. For this reason, in the present study the days 0 and 42 were selected to represent the acute and recovery phase respectively. As hypothesized, the pigs fed the low-CP diet and low AM/AP ratio had a lower diarrhea index in the first week postweaning compared with a conventional low-CP diet. However, the same reduction was not observed when comparing the low AM/AP and low-CP diet with the high CP diet, suggesting that the observed effect cannot be directly related to a decrease in the fermentation of undigested dietary protein in the large intestine. Instead, it is more likely attributed to a nutrient imbalance, as suggested by Rocha et al. (2022). This assumption is further supported by the absence of any significant effect of the diet on the fecal ammonia. Moreover, no acute gut inflammation was observed in the present study, indeed, the fecal concentration of calprotectin did not differ among the groups of pigs. Calprotectin is a protein complex released by activated neutrophils which are immune cells involved in the inflammatory response. During inflammation in the gastrointestinal tract calprotectin levels increase in the feces (Lallès and Fagerhol, 2005).

On the other hand, the observed reduction in diarrhea in the current study might be associated with a direct modulation of the gut microbiota. This is supported by the fact that the fecal bacterial diversity in the diet with low CP and AM/AP ratio was increased on day 10 together with a high relative abundance of *Prevotella*, without impacting the concentration of SCFAs. The increase in *Prevotella* agrees with the results of Zhou et al. (2022) in which the use of a diet with a low AM/AP ratio (~0.05) significantly increased the abundance of *Prevotella* in the feces compared with other higher ratios. *Prevotella*, is a genus commonly harbored in pig gut and plays a significant role in host physiology. This symbiotic relationship with the host has been linked to enhanced feed efficiency, immune system development, and overall health in pigs (Amat et al., 2020). Since AP is quickly digested in the pig’s small intestine, it is less fermentable by gut bacteria. However, intermediate products of enzymatic hydrolysis of AP can exert a prebiotic effect, leading to an increase in both the abundance and diversity of bacteria (Zhang et al., 2020). Moreover, the higher microbial diversity evidenced in pigs fed a low AM/AP indicates a more resilient and stable gut microbial ecosystem as suggested by the presence of a wide range of microbial species with various metabolic capabilities. This diversity ensures that the gut microbiota can adapt to changes in diet, environmental conditions, and challenges, maintaining stability and functionality. Finally, a higher microbial diversity ensures a higher functional redundancy within the gut microbiota. Functional redundancy means that multiple microbial species possess similar functional capabilities; if one species is compromised or lost, other species with similar functions can compensate and maintain the overall ecosystem functionality (Guevarra et al., 2019).

Furthermore, an excessive reduction in CP in pig diets can hinder the production of SCFAs by the microbiota, because bacteria need a minimum nitrogen level for fermentation. The only observed effect on SCFAs was a decrease in the isobutyrate concentration in the LP diet. Isobutyrate is produced during the fermentation of branched-chain amino acids (**BCAAs**). A decrease in isobutyrate concentration in an LP diet suggests that reducing dietary protein intake might decrease protein fermentation in the gut, as there would be fewer BCAAs available for microbial fermentation (Zhou et al., 2016).

In conclusion, whereas reducing dietary CP had a negative effect on growth performance, reducing the AM/AP reduced the diarrhea and increased gut bacterial diversity. The present results would encourage further studies on the optimization of the AM/AP ratio in weaned pigs fed diets rich in crystalline AAs, potentially improving both their gut health and overall performance. Finally, it can be desirable, considering the level of minerals and vitamins in low-CP diets to exclude side effect of the protein restriction (e.g., low protein concentrate) in meeting all the nutrient requirements of weaned pigs.

## Supplementary Material

skae370_suppl_Supplementary_Material

## Abbreviations

AAs: amino acids
ADG: average daily gain
AM: amylose
AM/AP: amylose–amylopectin ratio
AP: amylopectin
BCAAs: Branched-chain amino acids
BW: body weight
CP: crude protein
CTR: Control group
FI: feed intake
G:F: gain-to-feed ratio
LP: low protein
LPLA: low protein low amylose–amylopectin ratio
SCFA: short-chain fatty acids
