# Supplementary material for: Reduction of amylose–amylopectin ratio in low-protein diets: impacts on growth performance and intestinal health in weaned pigs
Source: J Anim Sci. 2024 Dec 9;102:skae370. doi: 10.1093/jas/skae370 (PMC11681340; doi:10.1093/jas/skae370)
Supplement: skae370_suppl_Supplementary_Material [file skae370_suppl_supplementary_material.docx]

Modulation of amylose-amylopectin ratio in low-protein diets: impacts on growth performance and intestinal health in weaned pigs

Federico Correa a, Diana Luise ^a^, Sara Virdis a, Clara Negrini a, Barbara Polimeni a, Roxana Elena Amarie ^c^, Andrea Serra ^c^, Giacomo Biagi ^b^, Paolo Trevisi ^a^

*^a^Department of Agro-Food Sciences and Technologies, University of Bologna, Bologna, Italy, 40127*

*bDepartment of Veterinary Science, University of Bologna, Ozzano dell’Emilia, Italy, 40064*

*^c^Department of Agricuture, Food and Environment, University of Pisa, Pisa, Italy, 56124*

Corresponding author: Paolo Trevisi. Email: [paolo.trevisi@unibo.it](mailto:paolo.trevisi@unibo.it)

**Supplementary Table 1.** Analysed protein and amino acids composition of the experimental diets.

| Item | Phase 1: d0-d13 | | | Phase 2: d14-d27 | | | Phase 3: d28-d49 | | |
| --- | --- | --- | --- | --- | --- | --- | --- | --- | --- |
|  | CTR | LP | LPLA | CTR | LP | LPLA | CTR | LP | LPLA |
| Crude protein | 18.3 | 16.3 | 16.1 | 16.7 | 14.4 | 14.7 | 16.8 | 14.3 | 14.5 |
| Sum of AAs | 16.8 | 15.1 | 14.82 | 15.64 | 13.43 | 13.55 | 15.78 | 13.48 | 13.48 |
| Total Lysine | 1.18 | 1.2 | 1.13 | 1.13 | 1.11 | 1.15 | 1.17 | 1.15 | 1.16 |
| Total Threonine | 0.8 | 0.73 | 0.72 | 0.76 | 0.69 | 0.64 | 0.77 | 0.68 | 0.67 |
| Total Methionine | 0.40 | 0.41 | 0.41 | 0.33 | 0.40 | 0.40 | 0.41 | 0.43 | 0.45 |
| Total Cystine | 0.33 | 0.30 | 0.29 | 0.28 | 0.25 | 0.25 | 0.28 | 0.24 | 0.24 |
| Total Tryptophan | 0.29 | 0.25 | 0.26 | 0.27 | 0.25 | 0.23 | 0.26 | 0.23 | 0.22 |
| Total Valine | 0.9 | 0.82 | 0.81 | 0.83 | 0.75 | 0.72 | 0.84 | 0.71 | 0.72 |
| Total Isoleucine | 0.65 | 0.57 | 0.56 | 0.6 | 0.47 | 0.48 | 0.6 | 0.47 | 0.47 |
| Total Leucine | 1.21 | 1.08 | 1.07 | 1.13 | 0.92 | 0.96 | 1.21 | 1.02 | 1.04 |
| Total Arginine | 0.88 | 0.78 | 0.78 | 0.88 | 0.7 | 0.73 | 0.97 | 0.78 | 0.77 |
| Total Phenylalanine | 0.79 | 0.69 | 0.67 | 0.73 | 0.58 | 0.59 | 0.73 | 0.6 | 0.59 |
| Total Tyrosine | 0.53 | 0.46 | 0.45 | 0.49 | 0.37 | 0.39 | 0.5 | 0.4 | 0.41 |
| Total Histidine | 0.37 | 0.34 | 0.34 | 0.36 | 0.3 | 0.31 | 0.4 | 0.33 | 0.33 |
| Total Serine | 0.83 | 0.72 | 0.71 | 0.74 | 0.61 | 0.62 | 0.75 | 0.63 | 0.63 |
| Total Alanine | 0.71 | 0.65 | 0.65 | 0.67 | 0.56 | 0.59 | 0.75 | 0.65 | 0.66 |
| Total Aspartic Acid | 1.23 | 1.05 | 1.04 | 1.2 | 0.86 | 0.91 | 1.36 | 1.04 | 1.02 |
| Total Glutamic Acid | 3.76 | 3.27 | 3.22 | 3.42 | 2.97 | 2.94 | 3.02 | 2.58 | 2.56 |
| Total Glycine | 0.67 | 0.6 | 0.6 | 0.65 | 0.55 | 0.57 | 0.67 | 0.57 | 0.57 |
| Total Proline | 1.29 | 1.14 | 1.12 | 1.18 | 1.09 | 1.07 | 1.1 | 0.97 | 0.97 |

^1^CTR= control group fed a standard diet with a medium-high CP content and high AM/AP (d0-d14: 18.0% CP, 0.13 AM/AP; d14-d28: 16.6% CP, 0.30 AM/AP; d28-d49: 16.7% CP, 0.15 AM/AP), LP= a group fed a low CP diet with high AM/AP, (d0-d14: 16.0% CP, 0.17 AM/AP; d14-d28: 14.7% CP, 0.17 AM/AP; d28-d49: 14.5%CP, 0.25 AM/AP), LPHA = a group fed a low protein and low AM/AP (d0-d14: 16.0% CP, 0.09 AM/AP; d14-d28: 14.7% CP, 0.05 AM/AP; d28-d49: 14.5%CP, 0.09 AM/AP)


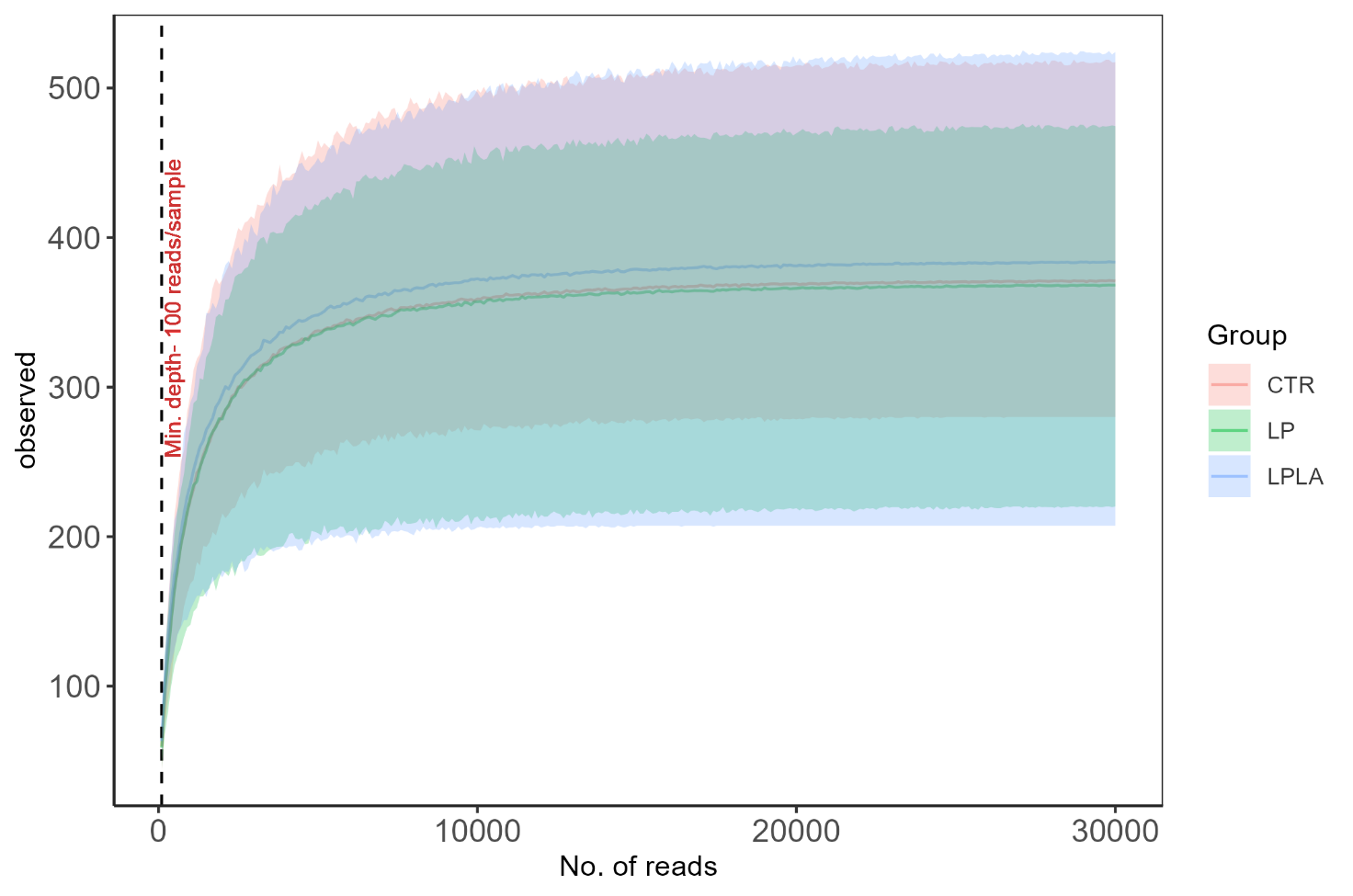


**Fig. 1.** Rarefaction curves of piglets’ faecal microbiota.

^1^CTR= control group fed a standard diet with a medium-high CP content and high AM/AP (d0-d14: 18.0% CP, 0.13 AM/AP; d14-d28: 16.6% CP, 0.30 AM/AP; d28-d49: 16.7% CP, 0.15 AM/AP), LP= a group fed a low CP diet with high AM/AP, (d0-d14: 16.0% CP, 0.17 AM/AP; d14-d28: 14.7% CP, 0.17 AM/AP; d28-d49: 14.5%CP, 0.25 AM/AP), LPHA = a group fed a low protein and low AM/AP (d0-d14: 16.0% CP, 0.09 AM/AP; d14-d28: 14.7% CP, 0.05 AM/AP; d28-d49: 14.5%CP, 0.09 AM/AP)

**Supplementrary Material.** R Codes used for the statistical model

# Load Libraries

library(car)

library(lme4)

library(emmeans)

library(microbiomeutilities)

library(microbiome)

library(knitr)

library(tibble)

library(dplyr)

library(plyr)

library(vegan)

library(ggpubr)

library(patchwork)

library(ggplot2)

library(microbiomeMarker)

# Statistical Model for Volatile Fatty Acids

# Volatile fatty acids

model <- lmer(log(VFA) ~ Diet + (1|Sow), data = data)

Anova(model)

pairs(emmeans(model, ~ Diet), type = "response")

# Statistical Model for ADG and BW

# ADG and BW

model <- lmer(ADG or BW ~ Diet + (1|Sow), data = data)

Anova(model)

pairs(emmeans(model, ~ Diet))

# Statistical Model for FI and FCR

# FI and FCR

model <- lmer(FI or FCR ~ Diet + (1|Sow), data = data)

Anova(model)

pairs(emmeans(model, ~ Diet))

# Microbiota Script

# Uploading files

ps <- readRDS(file = "ps_psr_waxy.rds")

sam_data <- read.table("sam_data.csv", sep=";", header = TRUE, row.names = 1)

ps@sam_data <- sample_data(sam_data)

# Subsetting samples by Timepoint

ps_waxy_T1 <- subset_samples(ps, Timepoint == "T1")

ps_waxy_T2 <- subset_samples(ps, Timepoint == "T2")

# Beta diversity analysis for T1 and T2

# Function to perform beta diversity analysis

analyze_beta_diversity <- function(ps_sample, title_suffix) {

ps_clr <- microbiome::transform(ps_sample, transform = 'clr')

vst_pcoa <- ordinate(ps_clr, method = "PCoA", distance = "euclidean")

eigen_vals <- vst_pcoa$values$Eigenvalues

plot_ordination(ps_clr, vst_pcoa, color = "Diet", shape = "Timepoint") +

geom_point(size = 2) +

labs(col = "Diet") +

coord_fixed(sqrt(eigen_vals[2] / eigen_vals[1])) +

ggtitle(paste("PCoA", title_suffix)) +

theme_test() +

scale_shape_discrete(name = "Timepoint", labels = c("d10", "d42"))

}

# Beta diversity T1 and T2

beta1 <- analyze_beta_diversity(ps_waxy_T1, " - d10")

beta2 <- analyze_beta_diversity(ps_waxy_T2, " - d42")

# Adonis Analysis

# Function to perform Adonis analysis

analyze_adonis <- function(ps_sample) {

metadata <- as(sample_data(ps_sample), "data.frame")

set.seed(25)

adonis.test <- adonis2(euc_dist ~ Diet, permutations = 999, data = metadata)

print(adonis.test)

}

# Adonis analysis for T1 and T2

analyze_adonis(ps_waxy_T1)

analyze_adonis(ps_waxy_T2)

# Alpha Diversity Analysis

# Function to perform Alpha diversity analysis

analyze_alpha_diversity <- function(ps_sample, comparisons, mycols, title_suffix) {

ps_rarefy <- rarefy_even_depth(ps_sample, sample.size = min(sample_sums(ps)), rngseed = TRUE, replace = TRUE, trimOTUs = TRUE, verbose = TRUE)

# Inverse Simpson

p.simp <- plot_diversity_stats(ps_rarefy, group = "Diet",

index = c("diversity_inverse_simpson"),

group.colors = mycols, label.format = "p.format", stats = FALSE)

p.simp <- p.simp + stat_compare_means(comparisons = comparisons, label = "p.format", tip.length = 0.05, method = "wilcox.test") +

ggtitle(paste("InvSimpson diversity index", title_suffix))

# Chao1

p.chao1 <- plot_diversity_stats(ps_rarefy, group = "Diet",

index = ("chao1"),

group.colors = mycols, label.format = "p.format", stats = FALSE)

p.chao1 <- p.chao1 + stat_compare_means(comparisons = comparisons, label = "p.format", tip.length = 0.05, method = "wilcox.test") +

ggtitle(paste("Chao1 diversity index", title_suffix))

# Shannon

p.shannon <- plot_diversity_stats(ps_rarefy, group = "Diet",

index = ("diversity_shannon"),

group.colors = mycols, label.format = "p.format", stats = FALSE)

p.shannon <- p.shannon + stat_compare_means(comparisons = comparisons, label = "p.format", tip.length = 0.05, method = "wilcox.test") +

ggtitle(paste("Shannon diversity index", title_suffix))

return(list(p.simp, p.chao1, p.shannon))

}

# Alpha diversity analysis for T1 and T2

alpha_diversity_T1 <- analyze_alpha_diversity(ps_waxy_T1, my_comparisons, mycols, " - d10")

alpha_diversity_T2 <- analyze_alpha_diversity(ps_waxy_T2, my_comparisons, mycols, " - d42")

# Combining plots

patch <- alpha_diversity_T1[[1]] / alpha_diversity_T1[[2]] / alpha_diversity_T1[[3]] |

alpha_diversity_T2[[1]] / alpha_diversity_T2[[2]] / alpha_diversity_T2[[3]]

patch + plot_annotation(tag_levels = 'A')

ggsave("Alpha_diversity.tiff", height = 12, width = 8)

# Lefse Analysis

# Function to perform Lefse analysis

run_lefse_analysis <- function(ps_sample, title_suffix) {

set.seed(1)

ps_clr_Genus <- phyloseq::tax_glom(ps_sample, taxrank = 'Genus')

mm_lefse2 <- run_lefse(

ps_clr_Genus,

wilcoxon_cutoff = 0.05,

group = "Diet",

kw_cutoff = 0.05,

lda_cutoff = 3,

bootstrap_n = 100,

taxa_rank = "Genus", multigrp_strat = TRUE)

pmm <- microbiomeMarker::plot_ef_bar(mm_lefse2) + theme_test() +

ggtitle(paste("Lefse Analysis", title_suffix))

return(pmm)

}

# Lefse analysis for T1 and T2

pmmT1 <- run_lefse_analysis(ps_waxy_T1, " - d10")

pmmT2 <- run_lefse_analysis(ps_waxy_T2, " - d42")

# Combining Lefse plots

patch_lefse <- pmmT1 / pmmT2

patch_lefse + plot_annotation(tag_levels = 'A')
